# Laboratory earthquake forecasting: A machine learning competition

**DOI:** 10.1073/pnas.2011362118

**Published:** 2021-01-25

**Authors:** Paul A. Johnson, Bertrand Rouet-Leduc, Laura J. Pyrak-Nolte, Gregory C. Beroza, Chris J. Marone, Claudia Hulbert, Addison Howard, Philipp Singer, Dmitry Gordeev, Dimosthenis Karaflos, Corey J. Levinson, Pascal Pfeiffer, Kin Ming Puk, Walter Reade

**Affiliations:** ^a^Geophysics Group, Los Alamos National Laboratory, Los Alamos, NM 87545;; ^b^Department of Physics and Astronomy, Purdue University, West Lafayette, IN 47907;; ^c^Department of Earth, Atmospheric and Planetary Sciences, Purdue University, West Lafayette, IN 47907;; ^d^Lyles School of Civil Engineering, Purdue University, West Lafayette, IN 47907;; ^e^Department of Geophysics, Stanford University, Stanford, CA 94305;; ^f^Department of Earth Science, La Sapienza Università di Roma, 00413 Rome, Italy;; ^g^Department of Earth Science, Pennsylvania State University, University Park, PA 16802;; ^h^Laboratoire de Géologie, Département de Géosciences, École Normale Supérieure, PSL University, CNRS UMR, 8538 Paris, France;; ^i^Kaggle, Google, LLC, Denver, CO 80301;; ^j^H2O.ai, 1010 Vienna, Austria;; ^k^Private individual, Athens 11364, Greece;; ^l^Private individual, Jacksonville, FL, 32207;; ^m^Department of Electrical Engineering, Rheinisch-Westfälische Technische Hochschule Aachen University, 52056 Aachen, Germany;; ^n^Private individual, Bethesda, MD 20817

**Keywords:** machine learning competition, laboratory earthquakes, earthquake prediction, physics of faulting

## Abstract

Earthquake prediction, the long-sought holy grail of earthquake science, continues to confound Earth scientists. Could we make advances by crowdsourcing, drawing from the vast knowledge and creativity of the machine learning (ML) community? We used Google’s ML competition platform, Kaggle, to engage the worldwide ML community with a competition to develop and improve data analysis approaches on a forecasting problem that uses laboratory earthquake data. The competitors were tasked with predicting the time remaining before the next earthquake of successive laboratory quake events, based on only a small portion of the laboratory seismic data. The more than 4,500 participating teams created and shared more than 400 computer programs in openly accessible notebooks. Complementing the now well-known features of seismic data that map to fault criticality in the laboratory, the winning teams employed unexpected strategies based on rescaling failure times as a fraction of the seismic cycle and comparing input distribution of training and testing data. In addition to yielding scientific insights into fault processes in the laboratory and their relation with the evolution of the statistical properties of the associated seismic data, the competition serves as a pedagogical tool for teaching ML in geophysics. The approach may provide a model for other competitions in geosciences or other domains of study to help engage the ML community on problems of significance.

Earthquake prediction, which requires determination of the time, location, and size of an event before it begins, has had a long and controversial history. Tremendous effort has been expended in pursuing it, with occasional glimmers of hope but ultimately, disappointing results, leading many to conclude that short-term earthquake prediction is at best infeasible and perhaps impossible. In Charles Richter‘s own words, “only fools, charlatans and liars predict earthquakes.”

With machine learning (ML), the earthquake science community has a new suite of tools to apply to this long-standing problem; however, applying ML to the prediction problem raises multiple thorny issues, including how to properly validate performance on rare events, what to do with models that seem to have strong predictive value but may not generalize, and how to handle the output of opaque ML methods. Despite these challenges, recent work has shown that progress on some aspects of the prediction problem is possible. For example, ML has revealed that the time remaining before an earthquake in the laboratory and particular types of tectonic earthquakes known as slow slip events can be anticipated from statistical characteristics extracted from seismic data (1–3). In the following, we present an overview of the laboratory earthquake prediction problem. We then describe the methods of the ML competition, its outcomes, and resulting insights into fault physics.

## Perspective: Earthquake Prediction and Forecasting

Earthquake prediction, the “when, where, and magnitude” of an upcoming event, relies on the identification of distinctive precursors that might precede a large earthquake (4). It is well established that earthquakes may be preceded by foreshocks and followed by aftershocks (4)—known as the “foreshock–mainshock–aftershock” sequence. Foreshocks occur during earthquake nucleation as the fault prepares for rupture and are thought to represent failure of small frictional patches at or near where the fault will ultimately rupture in a mainshock. Numerous researchers have studied precursors in the laboratory (5–13), in simulations (14–16), and in the Earth (17–26). While seismic precursors are often observed in laboratory studies and in simulation, they are not reliably observed in the Earth (19, 23), with a few notable exceptions where slow earthquakes have been observed prior to large subduction earthquakes (27–31).

Indeed, in the early 1990s, the International Association of Seismology and Physics of the Earth’s Interior solicited information for a list of significant precursors (32). After intensive review of the scientific literature, the International Commission on Earthquake Forecasting for Civil Protection concluded in 2011 that there was “considerable room for methodological improvements” (32), an understatement to be sure. The report noted that many instances of reported precursors are contradictory and unsuitable for rigorous statistical evaluation. Published results are biased toward positive outcomes, and therefore, the rate of false negatives, where an earthquake exhibits no precursory signal, is unclear (32). The rate of false positives for proposed precursory phenomena is also rarely quantified.

Moreover, there exists a broad and contentious discussion regarding the nature of fault rupture—whether earthquakes are predictable or not (4, 18). If faults slip in an entirely deterministic manner, prediction well in advance of an earthquake may be possible; if they slip in a stochastic manner, forecasting immediately preceding failure may be possible but not long before.

In summary, we are a long way from earthquake prediction and forecasting, yet recent work focused on laboratory quakes offers a glimmer of hope.

## Recent Applications of ML in Earthquake Science

ML applications in geoscience have expanded rapidly over the last two decades. The confluence of new ML algorithms, fast and inexpensive graphical processing units and tensor processing units, and the availability of massive, often continuous datasets has driven this revolution in data-driven analysis. This rapid expansion has seen application of existing and new ML tools to a suite of geoscientific problems (33–36) that span seismic wave detection and phase identification and location (34, 37–45), geological formation identification (46, 47), earthquake early warning (48), volcano monitoring (49–51), denoising Interferometric Synthetic Aperture Radar (InSAR) (50, 52, 53), tomographic imaging (54–57), reservoir characterization (58–60), and more. Of particular note is that, over the past 5 y, considerable effort has been devoted to using these approaches to characterize fault physics and forecast fault failure (1–3, 13, 35, 61–63).

## A Brief Sketch of ML

There are two broad categories of ML—"supervised” and “unsupervised” learning (some would argue that “reinforcement” learning is a third category) (38, 64).

Supervised learning describes a class of problems that involve teaching a model how to approximate a hypothetical function that maps input data to output data based on a number of input–output examples. After it is trained, the model is used to make predictions on test input data that the model has not seen before and with no knowledge of the target for the test data. This problem can be formulated either as a classification or as a regression. Regression is a familiar supervised learning approach that involves the prediction of a continuous label. Classification is a supervised learning problem that involves prediction of a class (a discrete target). Both classification and regression problems may have one or more input data variables of any dimension that may be any data type, such as numerical, time series, or image.

Unsupervised learning describes a class of problems that use an ML model to describe or extract relationships in data. Unsupervised learning operates upon only the input data without outputs or target. In the following, we describe advances toward earthquake prediction through the lens of supervised learning.

## ML Advances Earthquake Prediction in the Laboratory

**Fig. 1.**
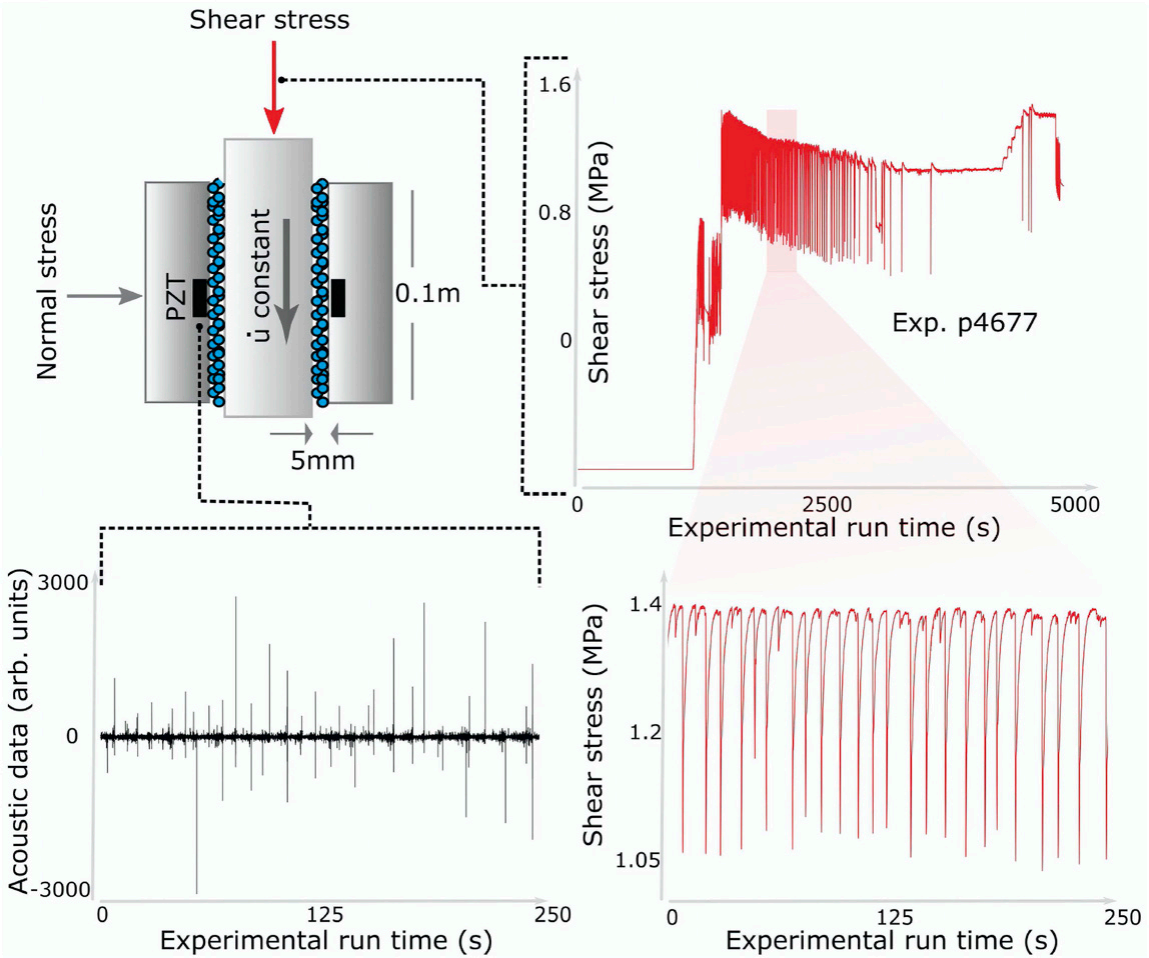
Experimental configuration and data collected. The biaxial experiment shear configuration and data collected. (*Upper Left*) The experiment is composed of three steel blocks with fault gouge contained within two shear zones. The fault gouge is composed of glass beads with dimensions on the order of 120 μm and a layer thickness of 4 mm for each fault. The configuration is held in place by a fixed horizontal (normal) load of 5 MPa. The central block is driven downward at a constant velocity. Acoustic emission is recorded by a lead zirconate titanate (PZT) piezoelectric ceramic transducer. (*Upper Right*) Measured shear stress as a function of experimental run time. There is a run-in displacement during which the shear stress increases until the gouge material begins to stick–slip quasiperiodically. This occurs for roughly half the experiment, followed by the central block failing intermittently and then sliding stably. *Lower Left* and *Lower Right* show expanded views of the acoustic emission signal and shear stress signal, respectively, for the shaded region in the shear stress signal, where the laboratory quakes are relatively periodic. arb., arbitrary. Reprinted with permission from ref. 1.

Earthquake forecasting with ML had its first verifiable success in the laboratory (1, 2) by analyzing the characteristics of a continuous seismic signal broadcast from a fault shear zone. The data were obtained from an experiment conducted in a double-direct shear geometry using a biaxial shear apparatus (7, 65–68) (Fig. 1).

In the experiment, two faults containing granular fault gouge were sheared simultaneously with a prescribed shear velocity and constant normal load. Mechanical data measured on the apparatus included the shearing block velocity, the applied load, the gouge-layer thickness, the shear stress, and friction. In addition, continuous records of fault zone seismic wave radiation were recorded with piezoceramics embedded in side blocks of the shear assembly (69, 70). The laboratory faults fail in quasirepetitive cycles of stick and slip that mimic to some degree the seismic cycle of loading and failure on tectonic faults (Fig. 1).

**Fig. 2.**
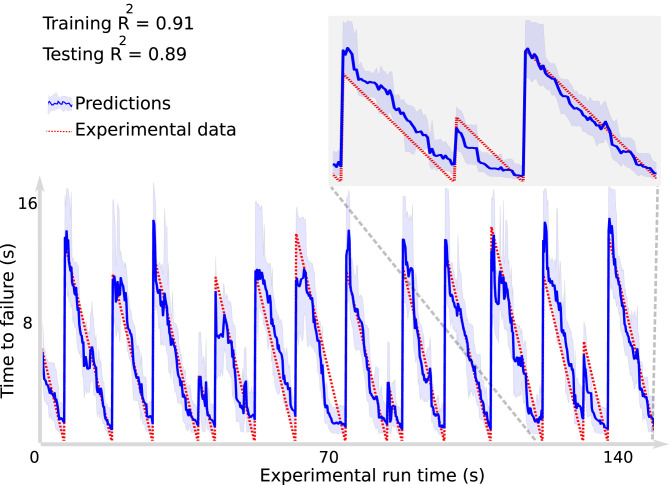
Random forest (RF) approach for predicting time remaining before failure. Failure times are determined by the large stress drops associated with the laboratory earthquakes, as seen in Fig. 1, *Lower Right*. The RF prediction (blue line) is shown on the testing data (data not previously seen by the ML algorithm) with 90% CIs (blue shaded region). The predictions agree very well with the actual remaining times before failure (red curve). The testing data are entirely independent of the training data and were not used to construct the model. *Inset* shows an expanded view of three slip cycles, illustrating that the trained model does well on aperiodic slip events (data are from laboratory experiment no. p2394 at Penn State). Reprinted with permission from ref. 1.

The approach uses a decision tree-based ML model, known as a random forest algorithm (71). The study relies exclusively on a snapshot of the continuous seismic signals recorded during shear (Fig. 2) to predict failure time (independent decision tree models were developed to predict the instantaneous fault shear stress, shear displacement, and gouge thickness). The problem, posed as a regression, uses the continuous recorded seismic data as input and the fault time to failure as the target (and respectively, the shear stress, displacement, and gouge thickness using independent ML models). The time to failure, used as ground truth, is calculated from the shear stress signal measured on the device. During training, the input and target data are used to construct the ML model. During testing, only the seismic data are seen by the model—the recorded shear stress is taken as ground truth and was not seen by the model during testing.

The model predicts the timing of laboratory earthquakes with high fidelity (Fig. 2). By applying a related decision tree ML model known as gradient-boosted trees (72), Hulbert et al. (2) can predict failure times for slow slip as well [a slow slip event is a “slow earthquake” that occurs in the laboratory (68) and in Earth (73, 74) and is a member of the spectrum of slip behaviors that range from fast (earthquakes) to very slow (75, 76)]. Hulbert et al. (2) are also able to predict slow earthquake magnitude using a separate ML model, albeit with less accuracy than for the earthquake timing. The ML model identifies why prediction was possible—the continuous seismic signal power evolves in a predictable manner throughout the stress cycle. This characteristic is used by the ML model to learn instantaneous and future characteristics of the fault system (e.g., see Fig. 4*C*). Other studies were also conducted with unsupervised approaches (77, 78).

Following analysis of the continuous wave seismic signals, a high-fidelity catalog of seismic signals was assembled from the large number of minute seismic precursor events occurring in the laboratory. The catalog feature characteristics are used to forecast future fault slip by applying a gradient-boosting model (63). The target is the time to failure, obtained from the measured shear stress on the sample. The study also shows that, as the earthquake catalog recording fidelity is decreased, predicting performance progressively decreases.

## ML Advances Slow Earthquake Prediction in Earth

**Fig. 3.**
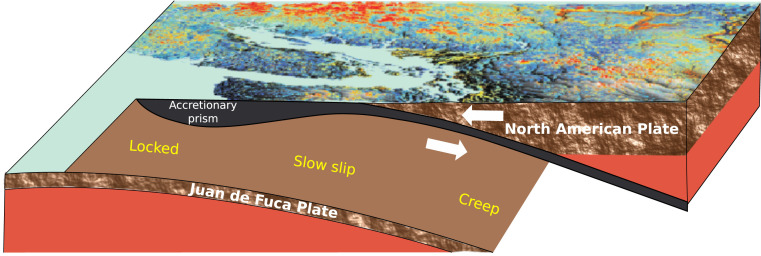
Subduction in Cascadia. Cross-sectional view of the Cascadia subduction zone in the region of Vancouver Island. Arrows indicate the sense of motion of the subducting Jan de Fuca plate beneath the North American plate. Adapted from ref. 61, which is licensed under CC BY 4.0.

The laboratory fault can be viewed as a single frictional patch and represents a relatively homogeneous system that is designed to facilitate the understanding of the underlying processes. A fault in the Earth, on the other hand, is composed of a very large number of frictional patches that behave as an ensemble when fault slip occurs and does so in the context of complex and heterogeneous Earth materials. These differences raise the question of how readily one can generalize from the laboratory to the Earth. The prediction approach devised from the laboratory data is scaled to Earth in the Cascadia subduction zone, where the Juan de Fuca tectonic plate subducts beneath the North American plate. Parts of the Cascadia subduction zone exhibit slip events with a duration on the order of weeks, occurring approximately every 14 mo, manifest by the North American plate slipping southwesterly over the Juan de Fuca plate (Fig. 3) (79, 80).

**Fig. 4.**
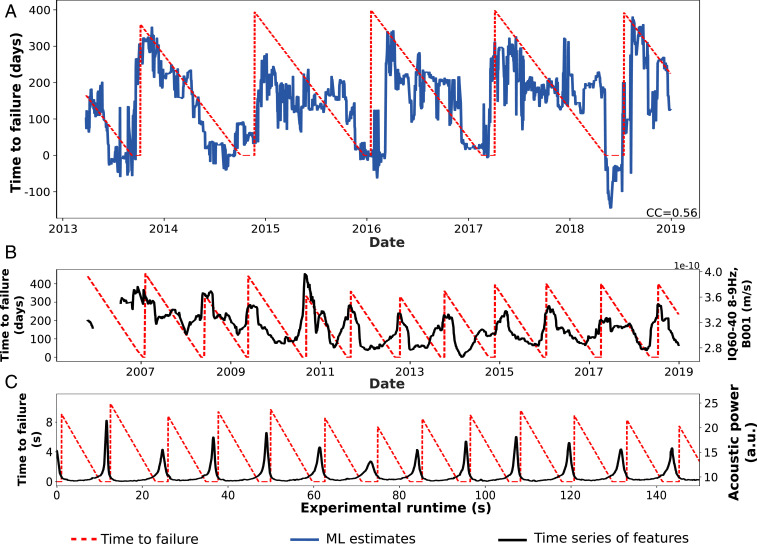
Slow slip time to failure estimates, seismic features identified by the ML model, and comparison with laboratory experiments. (*A*) Testing set ML estimates of time to failure (blue) and measured time to failure of slow earthquakes in Cascadia, in the region of Vancouver Island. CC, correlation coefficient. (*B*) The most important statistical feature of the seismic data is related to seismic signal energy (black curve). The seismic feature shows systematic patterns evolving through the slip cycle in relation to the timing of the succeeding slow earthquake in Cascadia (left axis). (The feature IQ60-40 range is the interquantile obtained by subtracting the 60th percentile from the 40th percentile.) (*C*) For comparison, the most important statistical feature found in laboratory slow slip experiments is acoustic power, which is proportional to signal energy (right-hand vertical axis). The similarity of the two measures, one in Earth and the other in the laboratory, suggests that the slip processes at both scales are related. Adapted from ref. 61, which is licensed under CC BY 4.0.

In the laboratory, slip characteristics are measured directly on the device. In Earth, slip takes place on the subduction interface at depth, all of the while emitting seismic signals. The fault displacement is measured indirectly at Earth’s surface using data from global positioning system stations. Estimates of the time remaining before the next slow slip event on the testing dataset are shown in Fig. 4 and are from ref. 61. This plot shows the ML slip timing estimates (in blue) and the time remaining before the next slow slip event (ground truth; dashed red lines). This ground truth is a countdown to the next slip event, similar to that developed for the laboratory data.

## Why Scientific Competitions?

Scientific challenge competitions were common in the 1800s in France to advance the understanding of light and how light interacts with materials (e.g., ref. 81). More recently, challenges such as the “XPRIZE” (https://www.xprize.org/) have been used since 1994 to promote the development of technology and methods related to spaceflight, learning, and oil cleanup. Competitions enable identification of the current state of the art, drive innovation, and attract engineers and scientists from vastly different disciplines, and potentially different scientific subcultures within disciplines, to develop novel solutions to proposed problems.

Since 2010, Kaggle (which was acquired by Google in 2017) has provided a platform for the ML world community that hosts competitions to propose and develop data analysis solutions for a wide range of problems. One notable example is the competition sponsored by NASA, the European Space Agency, and the Royal Astronomical Society to detect dark matter through gravitational lensing (https://www.kaggle.com/c/mdm). Surprisingly, this competition was won by a glaciologist who used techniques that had been developed for detecting edges in glacier fronts from satellite images.

A major idea driving the Kaggle philosophy is to facilitate the linking of data science and domain expertise for the effective application of ML approaches to challenging scientific problems.

The retrospective analysis of the laboratory and Cascadia datasets demonstrates that information on the state of a fault is imprinted in continuously generated signals. This raises the question of what other information may be contained in a signal that could have predictive value for time to failure in the Earth. With that in mind, we created a competition with the goal of attracting worldwide attention and to generate new ideas for addressing the challenge of earthquake prediction. Our aim was to learn about novel ML approaches that may help with earthquake prediction and also to attract ML-centered talent to Earth-related problems. In the next section, we describe the details and outcomes of the competition.

## The Kaggle Competition

The competition posed the question: can ML extract informative signatures from a small portion of continuous seismic data to predict the timing of upcoming laboratory earthquakes? The data were collected with the same biaxial device described in Fig. 1 but for conditions near the frictional stability transition where laboratory quakes exhibit complex metastable slip behavior (82). In particular, the laboratory earthquake data exhibited less periodic failures in contrast to the experiments described previously. Aperiodic failures are more difficult to predict, especially during the initial stages of the earthquake cycle. Predictions typically improve as failure approaches, as was shown in Fig. 2.

**Fig. 5.**
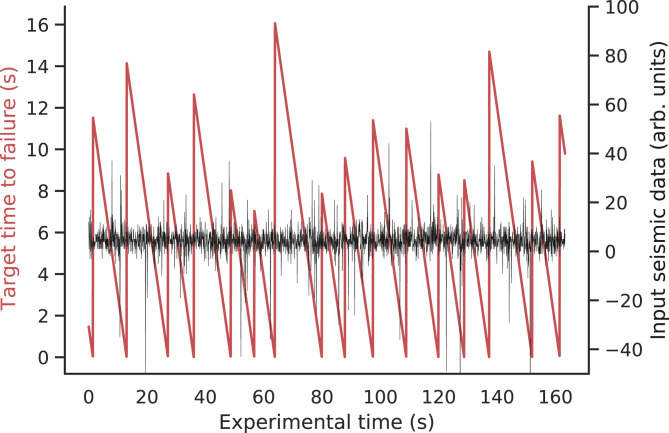
Competition training data. The black curve shows the seismic signal recorded on a piezoceramic transducer located on the biaxial apparatus side block (apparatus is shown in Fig. 1). Each burst in amplitude corresponds to a laboratory earthquake. The red curve shows the time to failure derived from the earthquakes and the measured shear stress on the experimental apparatus (as in Fig. 1). Competitors were tasked with predicting the time before the next laboratory quake only based on a small snapshot of seismic data.

The training data comprised a single continuous time segment of the recorded seismic data exhibiting multiple laboratory earthquakes (Fig. 5). The test data consisted of individual small segments of a different portion of the same experimental data. Thus, the predictions from the test data could not be assumed by contestants to follow the same regular pattern seen in the training data, making the prediction challenging. There was no overlap between the training and testing sets.

## The Kaggle Competition Outcome

**Fig. 6.**
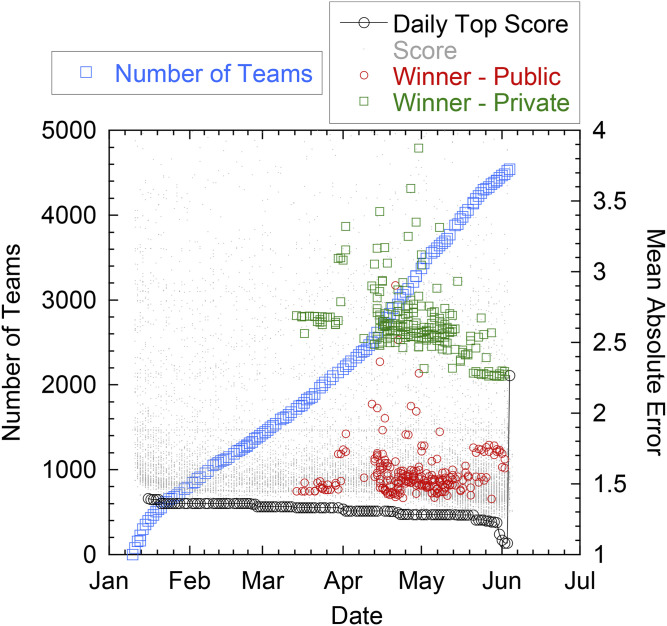
Evolution of MAE scores. The number of teams (light blue squares) and the value of the MAE of the daily first place team on the validation set (black circles) over the period of the competition as determined on the public validation set until early June and finally determined on the private test set for the final ranking (hence the jump in MAE for the final evaluation). The gray dots represent MAE values for all other submissions on each day. The public (red circles; validation set) and private (green squares; testing set) MAEs for the winning team are shown for the winning team's submissions.

The Kaggle competition was announced at a special workshop that we organized on ML in geoscience held at the December 2018 Neural Information Processing Systems Conference in Montreal and the following week at a special session on ML in Geoscience at the 2018 American Geophysical Union Fall Meeting in Washington, DC. The competition was officially launched on 10 January 2019 and ended on 3 June 2019. The competition attracted 4,521 teams with over 5,450 competitors, the largest number of researchers working simultaneously on the same geophysical dataset ever, to our knowledge (Fig. 6). Over 59,890 entries were submitted to compete for monetary prizes (a total of US $50,000) awarded to the top five teams.

Each day, competitors/teams were allowed to submit a maximum of two solutions/methods to test their ML approach. Competitors made predictions on the entire test set each time, but only the resulting score from a validation subset (13% of the testing data) was revealed to the teams during the competition. Results of the validation set were posted on a “public leaderboard” for all to see. The model prediction scores from the remaining approximately 87% of the test data were kept hidden from the participants (“private leaderboard”), and at the conclusion of the competition, the scores from this portion of the data were used to obtain the final standings (which could be, and were, different from the public leaderboard scores) (see Figs. 6 and 9).

**Fig. 7.**
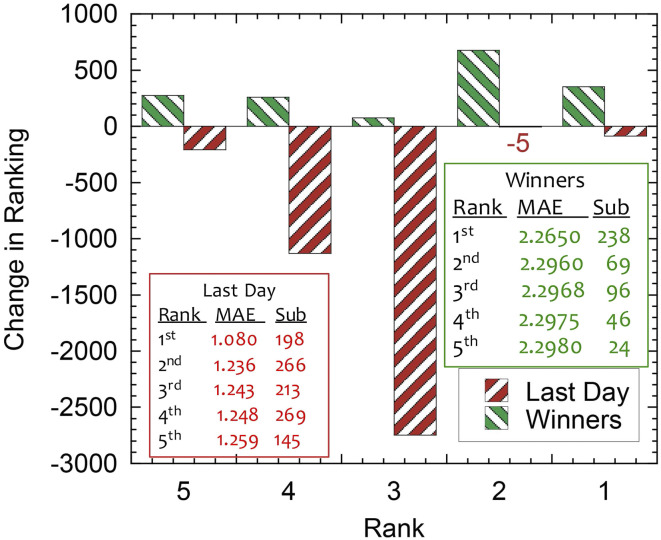
Comparison of the change in rank for the top five competitors on the last day of submission with that of the top five winners. Tables provide the rank, MAE, and total number of submissions for the top five competitors on the last day and for the winners.

For each submission, the Kaggle platform provided a score based on the mean absolute error (MAE; a measure of distance between the ML prediction and the testing data) and used the MAE on the validation set to rank the competitors. Six days into the competition, the MAE of the daily first place team was 1.396 and dropped to 1.080 on the day prior to closure of the competition (Fig. 6 has a summary). When the ML entries were run on the private leaderboard data, the MAE increased to 2.2650, with the result that some of the daily top teams dropped in the rankings (Fig. 7), a telltale sign of overfitting. The large gap between training and testing performance can be explained in part by the nonstationarity of the data (some physical properties of the experiment slowly evolved over time) that heavily penalized overfitting (more on that in the next section).

The evident camaraderie among the participants was surprising and gratifying—approaches were posted and discussed daily on the discussion board. Participants aided one another to improve their respective results. Indeed, this kind of collaborative work is often the norm in ML research, where codes are shared on, for example, GitHub and can readily be built upon by “forking,” in contrast to research in the natural sciences, where methods are described in detail in publications but often not shared directly.

The winning team, named “The Zoo,” comprised eight members from the United States and Europe, with expertise in mathematics, computer science, operations research, and electrical engineering. The members of Team Zoo have diverse backgrounds such as energy, credit risk, hotels, insurance, and Artificial Intelligence (AI). Many of the team members had not previously worked together. One member had experience working with signals from electroencephalogram research, while others had previous Kaggle experience (e.g., Walmart Recruiting: Trip Type Classification, Springleaf Marketing Response, Allstate Claims Severity) or worked professionally in ML. The diverse backgrounds of Team Zoo underscore the diversity of participants and demonstrate that the goal of assembling new perspectives and divergent experience and disciplines to the field of earthquake prediction was achieved through the competition. Team Zoo submitted a total of 238 entries and climbed 355 places to reach first place (Fig. 7). Interestingly, Team Zoo never achieved a daily first place ranking on the public leaderboard during the competition (Fig. 6). The winning teams that ranked second to fifth had very similar results, with differences in MAE of about 0.001 (Fig. 7). Team Zoo, however, had a large performance advantage of 30 times this small gap over other winning teams. Here, we will briefly describe the approach taken by the various teams.

**Fig. 8.**
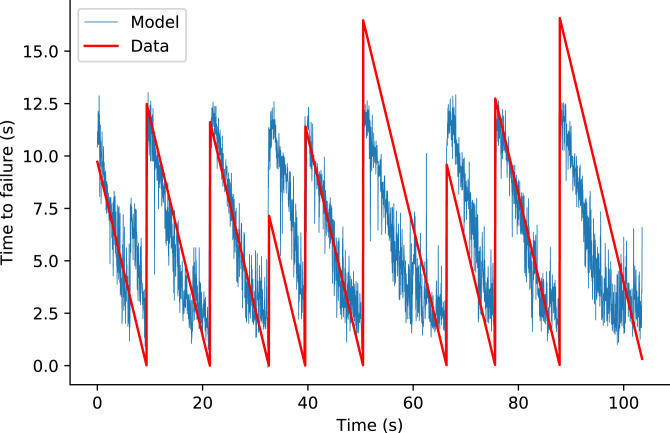
Winning model of the competition, by Team Zoo, on the test set. Red indicates time remaining before the next laboratory earthquake, as the experimental time progresses. Blue indicates predictions of Team Zoo’s winning model (an ensemble model of gradient-boosted trees and neural networks) based on small snapshots of seismic data (https://www.kaggle.com/dkaraflos/1-geomean-nn-and-6featlgbm-2-259-private-lb has additional details).

Team Zoo generated hundreds of features based on the original signals, denoised signals, and spectra. The four most important features used in their approach were 1) number of peaks in the denoised signal, 2) 20 percentile on the standard deviation (SD) of a moving window of size 50 points, 3) 4th Mel Frequency Cepstral Coefficient (MFCC) mean, and 4) 18th MFCC mean. MFCC is often used in speech processing for nonstationary signals. The diversity of these measures provides an example of how the competition resulted in new approaches. Their final approach included a blend of Light Gradient Boosting Machine (LGBM) and a neural network model fitted on threefold shuffled cross-validation (Fig. 8). Using this method, the team found that using “time-since-failure” as an additional loss improved model training. Most importantly, before feature calculation, their approach entailed adding noise to the data and training their models on a subset of the training data that have similar feature distribution compared with the test data (based on Kolmogorov–Smirnov tests). In doing so, Team Zoo effectively used the test (private) data as an additional validation set. Further, noise was added to allow for features that rely on median values and to allow for the removal of the median instead of the mean from each sample for better generalization. (Median removal is generally more robust to outliers than mean removal.) A full description of their approach can be found at https://www.kaggle.com/c/LANL-earthquake-Prediction/discussion/94390.

**Fig. 9.**
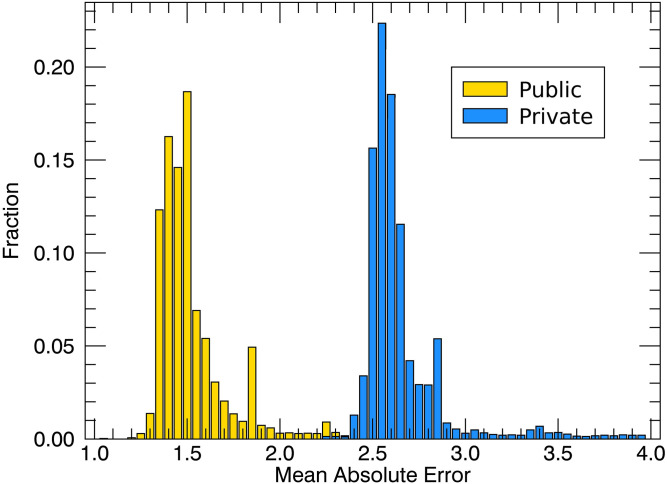
Distribution of MAE for all of the teams. Model performance of all of the competing teams on the two test sets (public and private). The performance dropped on the private set, a telltale of overfitting.

With a PhD in physics and currently working on the power spectrum of galaxy distributions, “JunKoda” (team of one) placed second by using CatBoost (an implementation of gradient-boosting trees) and 32 features. JunKoda found that the most important feature was the SD in the signal amplitude after the large-amplitude peaks were removed and that it was also important to avoid using means, as the data were not stationary. Similarly to Team Zoo’s approach of restricting training data to a distribution similar to test input data, JunKoda modified the training set based on figures from previously published work.

Team “Character Ranking,” with backgrounds in mathematics and law and with work experience in information security, cartoon publishing, and mobile games, placed third. This team found that using LGBM with many features performed slightly better than a recurrent neural network (RNN) approach with six features and simple gated recurrent unit. The two team members tried, independently, 1,900 and 2,400 features but found that by using only 2 features of the RNN, they could achieve a score of 2.3273 on the private leaderboard, showing that simpler models generalize better. In the end, they used a blend of both of their LGBM methods and the RNN.

Team “Reza,” composed of an electrical engineer, came in fourth with an LGBM model that included hyperparameter tuning and the selection of features based on the Kolmogorov–Smirnov test between the training and test datasets and again only used earthquake events that were similar to the test set, similar to Team Zoo’s approach. Selected features included moving SD/variance, moving skew/kurtosis, moving moments 5 and 6, autocorrelation, threshold detection, and peak detection. Team Reza found that most selected features were highly correlated with the target (time to failure). Team Reza developed 63 different LGBM algorithms over 63 different combinations of earthquakes in the training set. All models were trained with the same set of features but different sets of earthquakes. The final result was a simple average of the predictions from the 63 models.

The fifth place team “GloryorDeath” from Seattle, WA used features from standard signal processing libraries, including Butterworth filters and wavelet decomposition. Removal of the largest-amplitude peaks from the signals was used. An arbitrary scaling factor of 1.05 was one of their hyperparameters. The features were used in a simple feed-forward neural net in the deep learning library pytorch. One key innovation to their solution that dramatically improved their results was using a scaling of the time remaining before failure to indicate the state of the system as opposed to an absolute time to failure. In other words, predicting how far along in the seismic cycle the laboratory fault system is turned out to be easier than predicting the specific time remaining before the next quake. Additional details can be found at https://www.kaggle.com/c/LANL-earthquake-Prediction/discussion/94484.

## What Did We Learn from the Kaggle Competition?

Previous work on seismic data from Earth (3) suggests that the underlying physics may scale from a laboratory fault to large fault systems in Earth. If this is indeed the case, improvements in our ability to predict earthquakes in the laboratory could lead to significant progress in time-dependent earthquake hazard characterization. The ultimate goal of the earthquake prediction challenge was to identify promising ML approaches for seismic data analysis that may enable improved estimates of fault failure in the Earth. In the following, we will discuss shortcomings of the competition but also key innovations that improved laboratory quake predictions and may be transposed to Earth studies.

The approaches employed by the winning teams included several innovations considerably different from our initial work on laboratory quake prediction (1). Team Zoo added synthetic noise to the input seismic data before feature computing and model training, thus making their models more robust to noise and more likely to generalize.

Team Zoo, JunKoda, and GloryorDeath only considered features that exhibited similar distributions between the training and testing data, thereby ensuring that nonstationary features could not be used in the learning phase and again, improving model generalization. We note that employing the distribution of the testing set input is a form of data snooping that effectively made the test set actually a validation set. However, the idea of employing only features with distributions that do not evolve over time is insightful and could be used for scientific purposes by comparing feature distribution between portions of training data, for example.

Perhaps most interestingly from a physical standpoint, the fifth team, Team Reza, changed the target to be predicted and endeavored to predict the seismic cycle fraction remaining instead of time remaining before failure. Because they did not employ the approach of comparing input distribution between training and testing sets as done by the first, second, and fourth teams, the performance impact from the prediction of normalized time to failure (seismic cycle fraction) was significant.

As in any level of statistics, more data are in general better and can improve model performance. Thus, had the competitors been given more training data, in principle scores may have improved. At the same time, there is an element of nonstationarity in the experiment because the fault gouge layer thins as the experiment progresses, and therefore, even an extremely large dataset would not lead to a perfect prediction. In addition, Kaggle keeps the public/private test set split in such a way as to not reward overfitting. No matter how large the dataset is, if a model iterates enough times on that dataset, it will not translate well into “the real world,” so the competition structure was designed to prevent that opportunity.

It is worth noting that the ML metric should be carefully considered. In Earth, it will be important to accurately predict the next quake as it approaches, but MAE treats each time step equally with respect to the absolute error making this challenging.

Individuals participate on the Kaggle platform for many reasons; the most common are the ability to participate in interesting and challenging projects in many different domains, the ability to learn and practice ML and data science skills, the ability to interact with others who are seeking the same, and of course, cash prizes. The astounding intellectual diversity the Kaggle platform attracted for this competition, with team representations from cartoon publishers, insurance agents, and hotel managers, is especially notable. In fact, none of the competition winners came from geophysics. Teams exhibit collective interaction, evidenced by the step changes in the MAE through time (Fig. 6), likely precipitated by communication through the discussion board and shared code.

The competition contributed to an accelerating increase in ML applications in the geosciences, has become an introductory problem for the geoscience community to learn different ML approaches, and is used for ML classes in geoscience departments. Students and researchers have used the top five approaches to compare the nuances of competing ML methods, as well as to try to adapt and improve the approaches for other applications.

## Data Availability

The competition dataset and binary data have been deposited in Kaggle (https://www.kaggle.com/c/LANL-Earthquake-Prediction/data).
